# Perspectives From a Regional Plastic Surgery Centre on Evidence for the Purported Link Between SGLT2 Inhibitors and Fournier's Gangrene

**DOI:** 10.3389/fsurg.2021.754101

**Published:** 2021-12-10

**Authors:** Luke Taylor, Omar Asmar, Anirban Mandal, Ascanio Tridente, Kevin Hardy, Kayvan Shokrollahi

**Affiliations:** ^1^Department of Upper Gastrointestinal Surgery, Royal Gwent Hospital, Newport, United Kingdom; ^2^Mersey Regional Centre for Burns and Plastic Surgery, Whiston Hosiptal, Liverpool, United Kingdom; ^3^Department of Critical and Intensive Care, Whiston Hospital, Liverpool, United Kingdom; ^4^Department of Diabetology and Endocrinology, Whiston Hospital, Liverpool, United Kingdom

**Keywords:** burns–pathology, surgery, reconstructive surgery, Fournier's Gangrene, SGLT2 inhibitors, plastic surgery, type 2 diabetes (T2D)

## Abstract

**Introduction:** The recent report issued by the MHRA indicating an association of Sodium glucose linked transporter type 2 (SGLT2) Inhibitors with the contraction of Fournier's Gangrene (FG), has been drawn with insufficient supporting evidence and without an adequately powered study to make any meaningful assertions or recommendations. We aimed to look specifically at the currently available dataset used to link SGLT2 Inhibitors to FG and highlight what conclusions or inferences can meaningfully be made, in particular the power of any study that would be required to make sensible conclusions.

**Methods:** World literature review of SGLT2 Inhibitors and FG was performed. With a subsequent 10-year review of cases of FG seen in a regional burns and plastics centre. Data was collected retrospectively from the coding department at Whiston Hospital for all patients with necrotising fasciitis. An electronic document management system was used to identify patients with FG specifically as well as their diabetes state and medication history.

**Results:** Seventy-eight patients were admitted with FG, of whom 32 had diabetes mellitus (DM). Of those with DM none was taking an SGLT2 Inhibitor, 17 patients were taking metformin, a further nine patients were taking a second line medication and 14 required insulin injections.

**Discussions:** DM is a known major risk factor for FG, which is clearly observed in our patient cohort. The risk of patients with DM developing FG is irrespective of the medication patients are taking. The current articles and reports published have little ground to claim an association between SGLT2 Inhibitors and FG and are missing the crucial message that needs to be conveyed to the public: that DM is a major risk factor for FG and patients suffering with diabetes need to be extra vigilant.

## Introduction

Sodium glucose linked transporter type 2 (SGLT2) largely mediates the reabsorption of glucose in the proximal tubules of the kidneys (1). Inhibition of SGLT2 leads to an increase in glucose excretion via the kidneys as well as a reduction in the levels of plasma glucose, all independently of insulin (2). Pharmacological agents that block the actions of SGLT2, termed SGLT2 Inhibitors, have recently become available for the management of Type 2 Diabetes Mellitus (T2DM) (3). Research into SGLT2 Inhibitors began back in the early 1980's with the first approval for use in treatment of type 2 diabetes mellitus (T2DM) in 2013 (4). There are four SGLT2 inhibitors currently licenced for use in the UK; Dapagliflozin, Empagliflozin, Canagliflozin and Ertugliflozin, as well as combinations of these drugs with other diabetic medications, see also Table 1 (5). These novel drugs have been associated with improved cardiovascular risk factors and slower progression of renal disease in diabetic patients, as well as promoting some weight loss (6, 7). However, they do have some common side effects, including urinary tract and genital infections; which patients with diabetes are already at increased risk of developing (8, 9).

**Table 1:** List of SGLT2 inhibitors currently licenced for use in the UK according to the NICE clinical guidelines.

**Drug name**	**Brand name**
Dapagliflozin	Forxiga, Edistride
Canagliflozin	Invokana
Empagliflozin	Jardiance
Ertugliflozin	Steglatro
Dapagliflozin/Metformin	Ebymect
Dapagliflozin/Metformin	Xigduo
Dapagliflozin/Saxagliptin	Qtern
Canagliflozin/Metformin	Vokanamet
Empagliflozin/Metformin	Synjardy
Empagliflozin/Linagliptin	Glyxambi
Ertugliflozin/Metformin	Segluromet
Ertugliflozin/Sitagliptin	Steglujan

*Brand names for each drug have also been listed (5)*.

The pathophysiology of T2DM puts patients at greater risk of developing urinary tract and genital infection. Factors such as glucosuria, immune dysfunction, raised oestrogen levels and increased adherence of bacteria to uroepithelium all contribute to this increased risk (10, 11). As SGLT2 Inhibitors lead to increased glucose excretion via the kidneys, exacerbating the pathophysiology that occurs in the urinary tract of a diabetic patient, it would be expected that there would be an increased incidence of genitourinary infections (12–14). In rare cases diabetic patients can develop a severe infection known as Fournier's Gangrene, a fulminant form of necrotising fasciitis affecting the groin, perineum and genital areas (15, 16). This condition can be life threatening and requires immediate surgical excision and debridement of infected tissue, with likely reconstruction needed in a specialist centre (17).

In recent months, the Medicines and Healthcare Products Regulatory Agency (MHRA) and the Food and Drug Administration Agency (FDA) have issued reports indicating an association of SGLT2 inhibitors with Fournier's Gangrene contraction (18–20). The MHRA has published that it has received six yellow card warnings (21) of SGLT2 Inhibitors associated with Fournier's Gangrene; four men and two women (19). While the FDA reported 12 recorded cases of SGLT2 Inhibitors associated with Fournier's Gangrene over a period of March 2013 to May 2018 (18, 22–24). These results are shown in Table 2. These reports are describing a possible causative link between patients taking SGLT2 Inhibitors and Fournier's Gangrene, however, there is no statistical evidence to support a correlation between the two variables. The overarching combined message from the MHRA and FDA is that of a warning to both patients and clinicians. The warning to patients states that upon experiencing symptoms of tenderness, redness or swelling of the genitals and surrounding area as well as having an associated fever they should seek immediate medical attention. The message to health care professionals states that any patient with the above described symptoms should be examined for Fournier's Gangrene and, if taking an SGLT2 Inhibitor, should be stopped immediately (18, 20).

**Table 2:** The number of cases of Fournier's Gangrene associated with the use of SGLT2 inhibitors reported from both the Medicines and Health Regulatory Agency and the Food and Drug Administration Agency.

	**No. cases**	**No. men**	**No. women**	**SGLT2 inhibitors associated**
MHRA	6	4	2	Not reported
FDA	12	7	5	Dapagliflozin, Canagliflozin, Empagliflozin

Since the initially published warning by the FDA, it subsequently conducted a descriptive cases series over from the time frame of 1 March 2013 to the 31 January 2019. The results from this study are shown in Table 3 (25). The study describes an additional 35 cases of FG in association with an SGLT2 Inhibitor from the initial 12 cases that prompted the warning in February 2019. The study also identifies a further 19 cases of FG that are associated with other diabetic medications. It is also worth noting that the study highlights the limitation of the inability to establish causality or incidence.

**Table 3:** Results of a descriptive case series carried out by the FDA in June 2019 after the initially published warning in February 2019 (25).

**No. cases**	**No. men**	**No. women**	**Age**	**Time on SGLT2 inhibitor**	**No. requiring surgery**	**No. complications**
55	39	16	33–87	5 days−49 months	55	21

Over 500,000 patients are currently estimated to have exposure to an SGLT2 Inhibitor in the UK (26), and ~1.7 million patients have exposure in the USA (18, 27). This represents a vast population size of ~2.2 million people, yet only 18 cases of Fournier's Gangrene have been published with an association to patients taking an SGLT2 Inhibitor, far below the numbers required to prove causation between two independent variables. There have been no other studies to show any association between the diabetes medication patients are taking with the development of Fournier's Gangrene. Diabetes itself is the major risk factor for contraction of Fournier's Gangrene (11, 16) and demonstrating or refuting a link between any specific medication is not possible without a very large sample size and comparison with other medications.

The vast majority of patients with Fournier's Gangrene across the North West of England and Wales are transferred to Whiston Hospital in order to receive specialist treatment and reconstruction by the regional Burns and Plastics Department. The aim of this study was to retrospectively analyse all patients with Fournier's Gangrene seen at a hospital with a regional burns and plastic service over the last 10 years, reviewing the specific diabetic medications each patient was taking prior to their admission, and to explore further reports of this potential link.

## Methods

### Literature Review

A review of world literature on the subjects of SGLT2 Inhibitors and Fournier's Gangrene was performed. With a subsequent 10-year review of cases of Fournier's Gangrene seen in a regional burns and plastics centre.

### Data Collection

A database of all patients coded for Necrotising Fasciitis admitted into Whiston Hospital between January 2008 and December 2018 was retrospectively generated. An electronic document management system (EDMS) was used to review the notes for each patient in order to collate only the patients with Fournier's Gangrene and also to identify their diabetes state. EDMS was also used to analyse the medication history of each patient to identify patients taking an SGLT2 Inhibitor, as well as all other diabetic medications.

### Data Analysis

Data was analysed using JMP® statistical software and GraphPad Prism 8® software was used to generate and edit all graphs.

## Results

Analysis of patient data showed that 78 patients were admitted to Whiston Hospital, either with Fournier's Gangrene or for surgical reconstruction post-debridement following an episode of Fournier's Gangrene between January 2008 and December 2018. Of these patients a total of 32 had diabetes mellitus (DM), either type 1 or type 2. The age range for these patients was 31–97 years old, with the median age being 64 (lower and upper interquartile ranges are 54 and 78 respectively).

**Figure 1:**
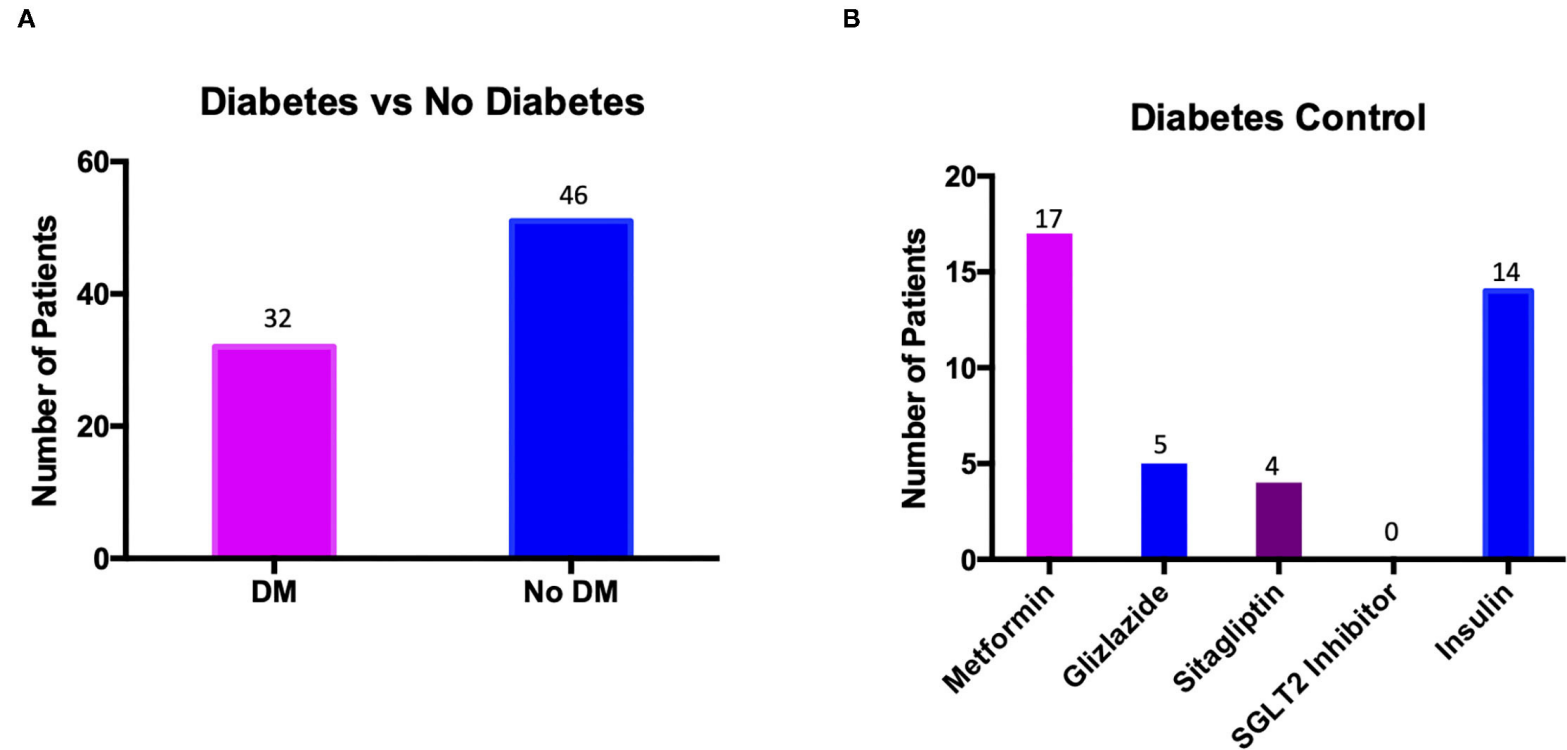
Graphs to show **(A)** the number of patients presenting with Fournier's Gangrene who had diabetes mellitus (DM) against the number who did not (No DM); **(B)** the number of patients with diabetes mellitus taking each individual medication.

As is seen in Table 2, 41.02% (*n* = 32) of patients with Fournier's Gangrene also had DM, of whom all were on medication in order to control their blood glucose levels. As per NICE guidelines some patients were taking more than one medication in order to control their diabetes. As shown in Table 4, 53.13% (*n* = 17) of patients were taking metformin, with a further 28.13% (*n* = 9) of patients requiring another second line medication to reach adequate diabetes control; breakdown of which medications were taken is shown in Figure 1. It was observed that no patients admitted with Fournier's Gangrene were taking an SGLT2 Inhibitor.

**Table 4:** Showing the percentages of patients with DM taking an SGLT2 inhibitor and the percentage of patients taking other 2nd line therapy for control of their diabetes.

**Number of patients**	**% of patients with DM**	**% DM patients taking SGLT2 inhibitor**	**% DM patients taking other 2nd line DM medication**	**% DM patients taking metformin**	**% DM patients taking insulin**
78	41.02	0	28.13	53.13	43.75

Figure 1A shows the number of patients presenting with Fournier's Gangrene who had DM (32 patients) and those who did not (46 patients). Figure 1B shows the breakdown of diabetes medication taken by the 32 patients with DM. Seventeen patients were taking metformin with a further nine patients requiring a second line treatment as well, either gliclazide or sitagliptin. A total of 14 patients required insulin for their diabetes control, either as primary treatment for T1DM (11 patients) or for add on therapy for T2DM (three patients). No patients were taking an SGLT2 Inhibitor for control of their diabetes.

## Discussion

Diabetes UK currently estimate that 4.6 million people are living with diabetes in the UK, 90% of whom are T2DM, with an increasing number of patients being diagnosed each year (28). There are ~500,000 patients in the UK currently taking an SGLT2 Inhibitor for diabetes control, therefore it stands that ~12% of all T2DM patients in the UK are taking an SGLT2 Inhibitor (26). Thus, it would be expected that approximately three to four patients in our dataset were taking an SGLT2 Inhibitor, however it was observed that none of the patients presenting with Fournier's Gangrene were taking an SGLT2 Inhibitor upon their admission into hospital.

Fournier's Gangrene is rare, and while we did not find any patients taking an SGLT2 Inhibitor who developed Fournier's, we did discover that they were taking a range of other diabetic medication. There is currently insufficient evidence in world literature to either support or refute an association of SGLT2 Inhibitors, or any diabetic medication in fact, with development of Fournier's Gangrene. It is clear, however, that the size of the study required to show correlation is undeniably large and will require collection of data on a national scale. Diabetes mellitus itself, rather than SGLT2 Inhibitors, is the major risk factor for development of Fournier's Gangrene, as supported by data from our own study with over 40% of patients having diabetes at the time of their hospital admission. It is known that diabetes mellitus predisposes patients to an increased risk of urinary tract and genital infections due to raised urine glucose content as well as reducing host immune factors (29, 30). When comparing SGLT2 Inhibitors to other second line T2DM medications, it was observed that there was no difference in the risk of urinary tract and genital infections between the two cohorts (31). These data suggest that all T2DM patients are at equal risk of developing these infections if their diabetes control is not adequate, irrespective of the specific medications they are taking. In fact, 28.13% of patients with T2DM who were admitted to Whiston Hospital with Fournier's Gangrene were requiring a second line medication (gliclazide or sitagliptin) on top of metformin, the first line medication given in T2DM as recommended by NICE (5). The requirement for second line diabetic medication in T2DM, such as an SGLT2 Inhibitor, therefore, could be a surrogate marker of poorly controlled diabetes. However, accurate HbA1c data would be required to show whether the significant risk of T2DM in patients developing Fournier's Gangrene appears to be poorly controlled diabetes rather than simply diabetes itself.

The only published negative effects of SGLT2 Inhibitors that have a statistical significance are the increased risk of diabetic ketoacidosis (DKA) and lower digital amputation, published in separate studies including over 17,000 patients currently taking an SGLT2 Inhibitor (31, 32). The current association between SGLT2 Inhibitors and Fournier's Gangrene published by the MHRA and FDA do not demonstrate correlation. This is due, in part, to the extremely small patient cohort and number of confounding variables. The suggested reports of SGLT2 inhibitors causing Fournier's Gangrene is at odds not only with our experience but the underlying case numbers and power of studies and reports simply cannot reasonably lead to the conclusions made. In order to do such would require a study size similar to that required for a phase 3 clinical trial of any pharmaceutical agent (33, 34). We have tentatively calculated the estimated sample size which may be required. The calculations make the following assumptions: we use the incidence in the male diabetic population of 1.6/100,000/year and we assume that a significant increase in the incidence in this population related to the use of SGLT2 Inhibitors would be 20%, increasing it to 1.92/100,000/year (male). We assume 10% of the sample would be lost to follow-up and 10% in each arm would cross over to the other arm. Assuming a 1:1 randomisation ratio, observation of 125,325,522 person-years would be required across the two arms (35). If we aimed to detect an increase in incidence rate of just 10%, all other assumptions remaining the same, observation of 478,515,626 person-years would be required across the two groups.

Our hospital data reconfirms diabetes mellitus as a significant risk factor for Fournier's Gangrene, with over 40% of patients having diabetes. Our study demonstrated that no diabetic patients in a regional centre with a catchment area of over five million patients who were admitted with Fournier's Gangrene were taking an SGLT2 Inhibitor. It is important to emphasise that this study in no way proves the absence of a link between Fournier's Gangrene and SGLT2 Inhibitors in exactly the same way that the studies already published suggestive of a link do not actually prove such a link exists (18, 19, 21–24). Our view is that in the absence of sufficiently powered studies the message from the MHRA is not that “cases of Fournier's Gangrene have been associated with SGLT2 Inhibitors and patients should seek urgent medical attention if experiencing any of the described symptoms” (36), rather the message should read “patients with diabetes mellitus in general, particularly if poorly controlled, should be extra vigilant to the symptoms of Fournier's Gangrene and indeed necrotising fasciitis anywhere in the body.” This broader vigilance still protects the SGLT2-taking diabetic population until such time as any causal relationship is proven with sufficiently powered studies.

## Conclusions

Diabetes mellitus (DM) is a major risk factor for Fournier's Gangrene, irrespective of the medication patients are taking. This makes it very difficult to link any diabetic medication to a relatively rare complication (Fournier's gangrene) without an enormous dataset and a sufficiently powered study. This is highlighted by the fact that in our regional service over a 10-year period not a single patient with Fournier's gangrene was taking an SGLT2 Inhibitor prior to their admission.

The recent messaging from MRHA and FDA with regards to risk of Fournier's Gangrene in patients taking SGLT2 inhibitors is more helpfully presented as a general awareness across the board of all diabetic patients and their risk of this devastating condition.

Our appraisal of our own data and what is currently available demonstrates that there is currently no evidence for a causal relationship between SGLT2 Inhibitors and Fournier's Gangrene other than the causal association between diabetes itself and Fournier's gangrene, and the published studies are lacking in power to prove such. In order to refute or prove a causal relationship, adequately powered studies that specifically take into account a patient's diabetes control, the use of diabetic medication (both first- and second-line treatments) as well as patient's compliance with their treatment is required. Our power calculation reveals that a 2-armed study of over 125 million patients would be needed to infer a significant increase in incidence related to exposure to the medications, and this may not be feasible. We are reassured by the absence of any patients in our catchment affected by this suggested link with SGLT2-inhibitors. Currently the most important message that needs to be conveyed to the public is that patients suffering with DM need to be extra vigilant for signs of Fournier's Gangrene regardless of their medication.

Robust statistical evidence does not exist that patients taking SGLT2 inhibitors are at greater risk of Fournier's gangrene either (a) because of that medication rather than their diabetes or (b) because of that medication as opposed to other more commonly consumed medications taken by diabetic patients. Prospective studies of considerable magnitude will be needed to demonstrate or refute any such association with any certainty, but it is clear from our appraisal that there appears more room for reassurance than for alarm.

## Data Availability Statement

The raw data supporting the conclusions of this article will be made available by the authors, without undue reservation.

## Author Contributions

LT performed data collection and analysis, created the figures, and was the primary author in writing the paper. OA performed data collection. AM provided feedback and contributed improvements for the design and write up of the paper. AT conducted statistical analyses for the paper and provided the power calculation required for future studies. KH provided expertise into the diagnoses of diabetes as well as the management and control of diabetes with regards to the medications reviewed in the paper. All authors contributed to the article and approved the submitted version.

## Funding

Funding for the paper was provided by the burns and plastics research fund for St Helens and Knowsley NHS Trust.

## Conflict of Interest

The authors declare that the research was conducted in the absence of any commercial or financial relationships that could be construed as a potential conflict of interest.

## Publisher's Note

All claims expressed in this article are solely those of the authors and do not necessarily represent those of their affiliated organizations, or those of the publisher, the editors and the reviewers. Any product that may be evaluated in this article, or claim that may be made by its manufacturer, is not guaranteed or endorsed by the publisher.

## Abbreviations

SGLT2: Sodium glucose linked transporter type 2
FG: Fournier's Gangrene
DM: Diabetes Mellitus
T2DM: Type 2 Diabetes Mellitus
MHRA: Medicines and Healthcare Products Regulatory Agency
FDA: Food and Drug Administration Agency
EDMS: Electronic Document Management System.
